# Combined Kernel-Based BDT-SMO Classification of Hyperspectral Fused Images

**DOI:** 10.1155/2014/738250

**Published:** 2014-08-27

**Authors:** Fenghua Huang, Luming Yan

**Affiliations:** ^1^Sunshine College, Fuzhou University, Fuzhou, Fujian 350015, China; ^2^College of Geographical Sciences, Fujian Normal University, Fuzhou, Fujian 350007, China

## Abstract

To solve the poor generalization and flexibility problems that single kernel SVM classifiers have while classifying combined spectral and spatial features, this paper proposed a solution to improve the classification accuracy and efficiency of hyperspectral fused images: (1) different radial basis kernel functions (RBFs) are employed for spectral and textural features, and a new combined radial basis kernel function (CRBF) is proposed by combining them in a weighted manner; (2) the binary decision tree-based multiclass SMO (BDT-SMO) is used in the classification of hyperspectral fused images; (3) experiments are carried out, where the single radial basis function- (SRBF-) based BDT-SMO classifier and the CRBF-based BDT-SMO classifier are used, respectively, to classify the land usages of hyperspectral fused images, and genetic algorithms (GA) are used to optimize the kernel parameters of the classifiers. The results show that, compared with SRBF, CRBF-based BDT-SMO classifiers display greater classification accuracy and efficiency.

## 1. Introduction

Hyperspectral remote sensing images have great spectral resolution and ground-object recognition capabilities because they have hundreds of fine and continuous wave bands. However, these images suffer from some problems, for example, the low spatial resolution, the high proportion mixed pixels, the huge amount of data, the uncertainty of data, and the Hughes phenomenon affecting the supervised classification. Hence, it is ineffective to classify based on spectral features alone. To improve the spatial resolution, hyperspectral images can be fused with high spatial resolution images, and the classification accuracy can be improved by combining spectral features with spatial features. The support vector machine is a well-established learning method that provides an approach to the traditional Hughes effect and overfitting problems. SVM has evident advantages and thus is widely used for classifying hyperspectral remote sensing images, especially for small samples, nonlinear, and high-dimensional pattern recognition problems. However, the traditional kernel function is a single kernel function. Single kernel-based SVM classifiers deliver lackluster performance in classifying combined spectral and spatial features. Multi-kernel SVM classifiers are more effective than single kernel classifiers because the former can combine kernel functions according to different classification features and improve classification performance by combining the advantages of spectral, spatial, and structural features. In this paper, a binary decision tree-based multiclass SMO (BDT-SMO) classifier is proposed to improve the classification accuracy and efficiency of hyperspectral remote sensing images. Two radial basis functions (RBFs) are employed for spectral and textural features and are then combined in a weighted manner to form a new combined radial basis function (CRBF). Eventually, the single radial basis function- (SRBF-) based BDT-SMO classifier and the CRBF-based BDT-SMO classifier are used, respectively, to classify hyperspectral fused images of land usages, and genetic algorithms are used to optimize the kernel parameters of classifiers. The results show that CRBF-based BDT-SMO classifiers have superior classification accuracy and efficiency.

## 2. Support Vector Machines (SVM)

**Figure 1:**
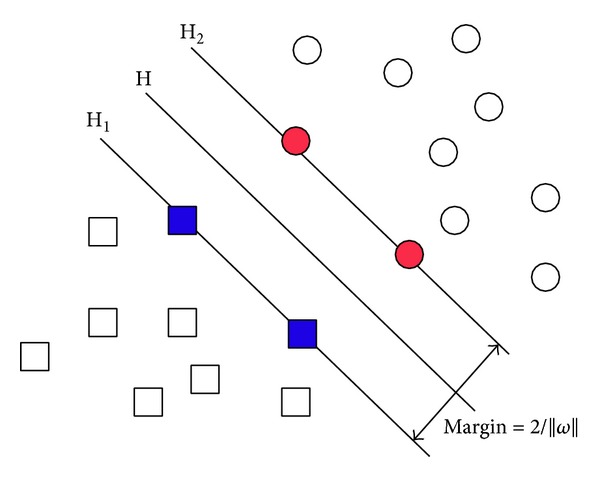
An example of the SVM optimal classification hyperplane.

SVM is a statistical learning-based machine learning method. The key idea behind an SVM is to take the Vapnik structural risk minimization (SRM) as the inductive principle [1]. If the feature space is linearly separable, the optimal classification hyperplane with low VC dimensions can be constructed in the high-dimensional feature space as the decision plane to maximize the distance between the two classes of data. Otherwise, kernel functions can be used to map the data into the high-dimensional feature space and then construct the optimal classification hyperplane in the high-dimensional feature space [1]. The optimal classification hyperplane has to be able to separate the two classes (the error rate is 0 during the training) and maximize the distance between the two classes [2]. An example of the SVM optimal classification hyperplane is shown in Figure 1 [3].

In Figure 1, circles and squares represent the two respective classes of samples. Let H be the optimal classification line. The points in the lines H_1_ and H_2_ parallel to H are support vectors, and the margin denotes the classification distance. Consider that for the classification plane *w* · *x* + *b* = 0, the following is true: *y*_*i*_[(*w* · *x*_*i*_) + *b*] − 1 ≥ 0, *i* = 1, …, *n*. In this case, the classification distance is 2/||*w*||. Maximizing the distance is equal to minimizing ||*w*||^2^. The classification plane that satisfies this condition is the optimal classification plane.

For the linearly separable sample set (*x*_*i*_, *y*_*i*_), *i* = 1, 2, …, *n*, *x* ∈ *R* ^*d*^, *y* ∈ {−1, + 1}, the Lagrange optimization method can be used to convert the classification plane optimization problem into a dual convex quadratic optimization problem. Hence, the linear sample classification problem is equal to the maximization problem of the following function [2]:

(1) $$\begin{array}{c} Q\left(a\right)=\overset{n}{\underset{i=1}{\sum }}a_{i}-\frac{1}{2}\overset{n}{\underset{i=1}{\sum }}\overset{n}{\underset{j=1}{\sum }}a_{i}a_{j}y_{i}y_{j}\left(x_{i}\cdot x_{j}\right), \end{array}$$

(2) $$\begin{array}{c} \text{s}.\text{t}.\;\overset{n}{\underset{i=1}{\sum }}y_{i}a_{i}=0,\;a_{i}\geq 0,\;\;i=1,\ldots ,n, \end{array}$$

where *a*_*i*_ denotes the Lagrange multiplier of each sample. This is a quadratic function optimization problem subject to the inequality constraints. In the solutions to (1), only a small subset of *i* is not equal to 0, and the corresponding samples are the support vectors. The classification plane is determined by the support vectors. That is, a small number of samples suffice to construct the optimal classification plane. Solving the above problem results in the following optimal classification function [3]:

(3) $$\begin{array}{c} f\left(x\right)=\mathrm{sgn}\left\{\overset{n}{\underset{i=1}{\sum }}a_{i}^{*}y_{i}\left(x_{i}\cdot x\right)+b^{*}\right\}, \end{array}$$

where *a*_i_* denotes the nonzero samples (i.e., support vectors) and b* is the classification threshold that can be computed using any support vector or is defined as the median of a pair of support vectors from both classes.

For nonlinear classification, nonlinear mapping (i.e., the kernel function) can be performed to map the input data from the original space, *R* ^*n*^, to the high-dimensional feature space, *Ω*, and then construct the hyperplane in the high-dimensional feature space [1]. In this case, a kernel function *K*(*x*_*i*_, *x*_*j*_) must be constructed in the original space and be made equal to the inner product of the transformed high-dimensional feature space. In this instance, the nonlinear classification problem is converted to the maximization of the following function [4]:

(4) $$\begin{array}{c} Q\left(a\right)=\overset{n}{\underset{i=1}{\sum }}a_{i}-\frac{1}{2}\overset{n}{\underset{i=1}{\sum }}\overset{n}{\underset{j=1}{\sum }}a_{i}a_{j}y_{i}y_{j}K\left(x_{i},x_{j}\right),\\ \text{s}.\text{t}.\;\overset{n}{\underset{i=1}{\sum }}y_{i}a_{i}=0,\;0\leq a_{i}\leq C\;\left(i=1,\ldots ,n\right), \end{array}$$

where *C* is a constant. Solving the above problem will result in the following classification criterion function [5]:

(5) $$\begin{array}{c} f\left(x\right)=\mathrm{sgn}\left\{\overset{n}{\underset{i=1}{\sum }}a_{i}^{*}y_{i}K\left(x_{i},x\right)+b^{*}\right\}. \end{array}$$

For nonlinear classification, choosing different inner kernel functions will produce different SVMs and have a different optimal classification hyperplane in the feature space. It has been shown that the common kernels are suitable for most nonlinear classification problems. The four common kernel functions are the linear kernel, the polynomial kernel, the radial basis function (RBF), and the sigmoid function [6]. The choice of SVM kernels and setup of the kernel parameters are largely dependent on empirical and experimental analysis, because no well-established methods are currently available for this. Foody and Mathur showed that the kernel parameters and the error penalty factor, *C*, rather than the class of the chosen kernels are the decisive factors in the performance of SVM [6].

Because traditional SVM algorithm is only suitable for two-class classification, the multiclass classification is often completed through the specific combination of multiple SVM classifiers. The multiclass classification methods commonly used are “one against one” SVM (OAO-SVM), “one against all” SVM (OAA-SVM), error correction code SVM (ECC-SVM), directed acyclic graph SVM (DAG-SVM), and binary decision tree SVM (BDT-SVM) [7].

## 3. Sequential Minimal Optimization (SMO)

**Figure 2:**
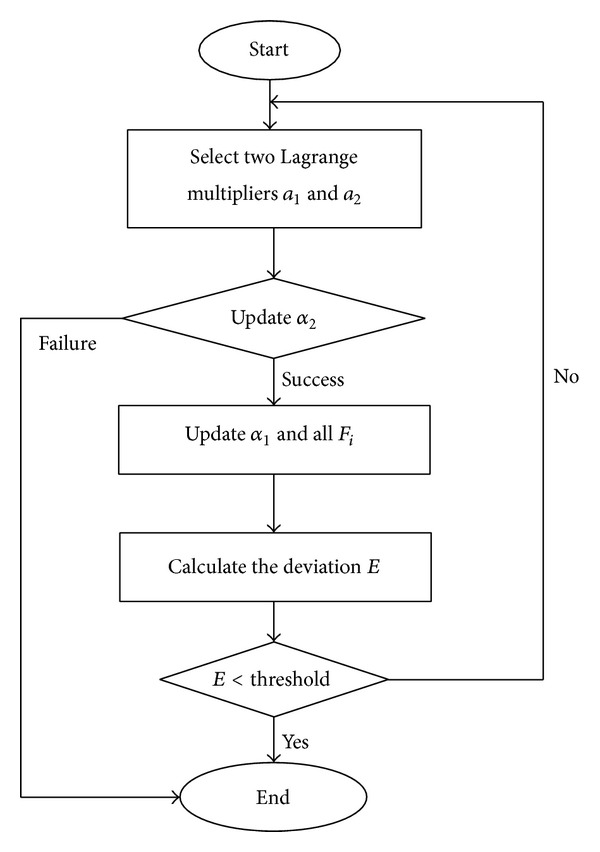
Procedures of the SMO training algorithm.

Traditional SVM algorithms can be boiled down to quadratic programming problems. Most existing approaches to the training of large-scale sample sets are based on decomposition and iteration, that is, solving the original quadratic programming problem by decomposing it into smaller quadratic programming problems. This method enormously increases computation complexity without guaranteeing accuracy [8]. The most prominent feature of SMO algorithms is that there is no need to run iterated algorithms because each small optimization problem can be solved analytically. Although the solution to each small optimization problem is not necessarily the final solution of the optimized Lagrange multiplier, the objective function approaches the minimal value [9]. The Lagrange multiplier can be minimized repeatedly until the KKT conditions are satisfied and the objective function is minimized. The procedures of the SMO training algorithm are shown in Figure 2 [8]. From Figure 2, it can be seen that SMO training involves firstly the determination of the two Lagrange multipliers *a*_1_ and *a*_2_ and the computation of *a*_2_'s upper bound, *H*, and lower bound, *L*. Secondly, *a*_2_ needs to be updated. If Δ*a*_2_ is smaller than the threshold, the updating fails. Otherwise, *a*_1_ needs to be updated according to relevant rules, and the values of all objective functions, *F*_*i*_, should also be updated. The deviation between the function's output and the target classification, *E*, will be computed. If *E* is smaller than the threshold, the algorithm terminates. SMO algorithms have been designed to solve two problems: firstly, they can solve simple optimization problems using analytical methods and secondly, they can choose strategies for optimizing the Lagrange multipliers [10]. Although SMO has more quadratic programming subproblems, it adopts the “divide and conquer” strategy. Its learning can therefore be performed in a parallel manner, enabling it to handle heavy matrix operations (e.g., inner product computation of the kernel functions) and sample searches. Hence, its processing speed is improved substantially. SMO algorithms can solve large-scale SVM training problems because they do not need to process large matrices and have no special requirements on memory space [10]. The SMO algorithm is also only suitable for two-class classification like traditional SVM algorithm, the multiclass classification is often completed through the specific combination of multiple SMO classifiers, and the multiclass classification methods commonly used are OAO-SMO, OAA-SMO, ECC-SMO, DAG-SMO, and BDT-SMO [7, 10].

## 4. Binary Decision Tree-Based Multiclass SMO (BDT-SMO) Classifiers

It was shown in [11–17] that both OAO-SVM and OAA-SVM have high classification speeds. When the data size is huge, however, the training efficiency will be downgraded severely and some regions are inseparable. Thus, they are unsuitable for classifying high-dimensional hyperspectral images that involve large amounts of sample data. ECC-SVM's generalization is independent of the dimensions of the features, but its coding schemes are highly subjective, because the problems of how to determine the code length, generate codes based on the minimum intersymbol Hamming distance, and search for the optimal arrangement have yet to be solved [11, 12]. The DAG-SVM model has a tree structure. Although it can classify quickly and provides an approach to the inseparable region problems, its classification efficiency is unsatisfactory when faced with many classes. In addition, the root nodes are mostly selected empirically, so improperly selected root nodes can directly reduce classification accuracy. BDT-SVM is similar to DAG-SVM, but the difference is that BDT-SVM only needs to construct *n* − 1 two-class SVM classifiers, whereas DAG-SVM needs *n*(*n* − 1)/2 internal nodes [13]. Additionally, each node in BDT-SVM has only one parent node and the internal node can be something other than a one-against-one classifier. In DAG-SVM, each node can have many parent nodes, and each internal node must be a one-against-one classifier [14]. BDT-SVM is characterized by high classification efficiency, fault tolerance, and generalization ability and thus it can produce better classification results than the other algorithms mentioned above [15].

An improvement on traditional SVM algorithms, SMO classification is faster than traditional algorithms for large sample datasets. SMO is therefore used in this work to replace traditional SVM algorithms, and the separation-based binary decision tree SMO (BDT-SMO) is employed for classifying hyperspectral remote sensing images. The existing researches [18, 19] have shown that, compared with OAO-SMO, OAA-SMO, ECC-SMO, and DAG-SMO, BDT-SMO has the best accuracy, efficiency, and generalization ability in the classification.

## 5. Multiple Radial Basis Kernel Function via Weighted Combination

### 5.1. Definition and Properties of Kernel Function

For the SVM nonlinear classification problem, the input data must be nonlinearly mapped in the original space, *R*_*n*_, to the linear classifiable data in the high-dimensional feature space using the kernel function, as well as constructing the classification hyperplane in the high-dimensional feature space. If a core function, *K*(*x*_*i*_, *x*_*j*_), satisfies the Mercer conditions, then *K*(*x*_*i*_, *x*_*j*_) corresponds to the inner product in a transformation space [20]. The Mercer theorem was proposed by Hilbert-Schmidt and it states the following: let **X** be a compact subset of **R** ^*n*^, for any given symmetric function, *K*(*x*_*i*_, *x*_*j*_), in **X** × **X** → **R**, if its integral operator in the Hilbert space satisfies the integral positive definiteness conditions [20]:

(6) $$\begin{array}{c} \forall f\left(x\right)\neq 0,\;\;\int f^{2}\left(x\right)dx<\infty ,\\ \iint K\left(x_{i},x_{j}\right)f\left(x_{i}\right)f\left(x_{j}\right)\;dx_{i}dx_{j}>0; \end{array}$$

then, there must exist a feature space **H** and a mapping *φ*(*x*) : **X** → **H**, and

(7) $$\begin{array}{c} K\left(x_{i},x_{j}\right)=\varphi \left(x_{i}\right)\cdot \varphi \left(x_{j}\right). \end{array}$$

The kernel function provides an effective approach to SVM nonlinear classification, and the inherent linearity of the high-dimensional space makes SVM more practically feasible. In various practical kernel applications, the kernel function operation in the original input space is equivalent to the high-dimensional operation executed after the nonlinear function is transformed, and the details of the nonlinear mapping are usually unknown. Different kernel functions satisfying the Mercer theorem can be combined as required by the application to construct a combined kernel function of higher complexity, which can be linear or nonlinear [21]. From the kernel function properties, it is known that if *K*_1_(*x*_*i*_, *x*_*j*_) and *K*_2_(*x*_*i*_, *x*_*j*_) are kernel functions in **R** ^*n*^ × **R** ^*n*^ and there exist constants *a*≧0 and *b*≧0, then the function *K*(*x*_*i*_, *x*_*j*_) in the following equations is also the kernel function [21]:

(8) K(xi,xj)=K1(xi,xj)+K2(xi,xj),K(xi,xj)=aK(xi,xj),K(xi,xj)=aK(xi,xj),

From the above equations it can be seen that the following equation will hold true if 0 ≤ *β*_*k*_ < 1, ∑_*k*=1_^*n*^ *β*_*k*_ = 1 and each kernel function *K*_*k*_(*x*_*i*_, *x*_*j*_) satisfies the Mercer conditions above [21]:

(9) $$\begin{array}{c} K\left(x_{i},x_{j}\right)=\overset{n}{\underset{k=1}{\sum }}\beta_{k}K_{k}\left(x_{i},x_{j}\right). \end{array}$$

### 5.2. Radial Basis Kernel Function

In theory, any function satisfying the Mercer theorem can be used as a kernel function, but different kernel functions must be designed for different applications. Common kernel functions include linear kernels, polynomial kernels, sigmoid kernels, and radial basis functions (RBFs). The first three functions are global kernels, and only RBF is a local kernel. Extensive work has shown that RBF-based SVM outperforms SVM based on the other three kernels and thus is used widely [22]. The inner product of the vectors *x* and *x*_*i*_ in the feature space for RBF is [23]

(10) $$\begin{array}{c} K\left(x,x_{i}\right)=\exp\left(-\frac{{||x-x_{i}||}^{2}}{2\sigma^{2}}\right), \end{array}$$

where *x*_*i*_ is a center point in RBF, ||*x* − *x*_*i*_|| is the norm of the vector *x* − *x*_*i*_ representing the distance between *x* and *x*_*i*_ [22], and *σ* is a width parameter of the function that controls the radial scope of the function and, that is, determines the width of the *x*_*i*_-centered range within which RBF exerts its influence. RBF is a highly local kernel, and the learning ability of RBF-based SVM is largely dependent on the parameter *σ* and the punitive factor, *C*. A small *σ* value means high SVM learning ability, low empirical risk in the structural risk, and poor generalization ability, giving rise to a large confidence range [24]. SVM uses the punitive factor, *C*, to balance empirical risk with confidence level to minimize the actual risk. A high value of *C* means a high degree of data fitting and poor generalization ability. Therefore, proper values of *C* and *σ* need to be selected to minimize structural risk and maximize generalization ability for classifiers [25].

### 5.3. Combined Radial Basis Kernel Function (CRBF)

There is some evidence [19, 23] that RBF can achieve high accuracy for spectral and textural feature classification and is capable of multiscale learning. To improve the accuracy and efficiency of spectral remote sensing classification, two different RBFs are firstly used for spectral and textural features and then perform weighted combination to form the CRBF. Consider [*x*_*i*_] as the feature vector of any pixel in the fused image, [*x*_*i*_^*s*^] as the combined vector of the pixel's spectral features, [*x*_*i*_^*w*^] as the pixel's textural feature vector; *x*_*i*_^*s*^ and *x*_*i*_^*w*^ can be combined in a separate or cross manner. The kernels of the two combination approaches are shown as follows, respectively:

(11) $$\begin{array}{c} K\left(x_{i},x_{j}\right)=\overset{m}{\underset{k=1}{\sum }}\lambda_{k}K_{k}\left(x_{i}^{s},x_{j}^{s}\right)+\overset{n}{\underset{k=1}{\sum }}\lambda_{k}K_{k}\left(x_{i}^{w},x_{j}^{w}\right), \end{array}$$

(12) K(xi,xj)=Ks(xis,xjs)+Kw(xiw,xjw)+Ksw(xis,xjw)+Kws(xiw,xjs).

Equation (11) shows the separate combination of multiple single-kernel RBFs, where *λ*_*k*_ is the weight and *m* and *n* are the number of spectral feature kernels and textural feature kernels, respectively. From the kernel properties, it can be seen that if 0 ≤ *λ*_*k*_ < 1 and ∑_*k*=1_^*n*^ *λ*_*k*_ = 1, then each kernel *K*_*i*_(*x*, *x*_*i*_) satisfies the Mercer conditions. Equation (12) shows the cross-combination of multiple single-kernel RBFs. If each kernel satisfies the Mercer conditions, it needs *x*_*i*_^*s*^ and *x*_*j*_^*w*^ in *K*_*sw*_(*x*_*i*_^*s*^, *x*_*j*_^*w*^) and *x*_*i*_^*w*^ and *x*_*j*_^*s*^ in *K*_*ws*_(*x*_*i*_^*w*^, *x*_*j*_^*s*^) to have the same number of dimensions. It is seldom used in practice due to the high computational complexity of the kernel. In this work, the spectral features have more dimensions than the textural features, so (11) is necessary for combinatorial classification of spectral and textural features. According to the classification features selected in this work, (11) is adjusted by using the same kernel *K*_*s*_(*x*_*i*_^*s*^, *x*_*j*_^*s*^) for spectral-terrain features. In addition, the same kernel *K*_*w*_(*x*_*i*_^*w*^, *x*_*j*_^*w*^) is used for different types of textural features (gray, gradient, and scale) to reduce the computational complexity of the kernel. The adjusted kernel *K*(*x*_*i*_, *x*_*j*_) is shown as follows:

(13) $$\begin{array}{c} K\left(x_{i},x_{j}\right)=\lambda \cdot K_{s}\left(x_{i}^{s},x_{j}^{s}\right)+\left(1-\lambda \right)\cdot K_{w}\left(x_{i}^{w},x_{j}^{w}\right). \end{array}$$

The CRBF constructed based on spectral and textural features is shown as follows:

(14) K(xi,xj)=λ·exp⁡(−||xis−xjs||2σs2)+(1−λ)·exp⁡(−||xiw−xjw||2σw2),

where *λ* is the weight (0 ≤ *λ* ≤ 1) and *σ*_*s*_ and *σ*_*w*_ are the standardization parameters determining the width of the range surrounding the center of the spectral radial kernel and the center of the textural radial kernel, respectively. The weight *λ* is used to balance the effect of the spectral radial kernel (SPRBF) and the textural weighted radial basis function kernel (WRBF) in CRBF. When *λ* is close to 1, SPRBF is dominant in CRBF; otherwise, WRBF is dominant. By adjusting *λ*, the weights of the effect of spectral and textural features in CRBF can be properly modified, and the optimum combinations to improve the learning and generalization abilities of CRBF can be identified.

## 6. DT-SMO Parameter Optimization Strategy Based on Genetic Algorithms

The types of kernels and selection of the corresponding parameters have enormous impact on the performance of the SVM classifiers. As mentioned above, the performance of the CRBF-based BDT-SMO classifier is dependent on the punitive factor, *C*, the weight, *λ*, and the standardization parameters, *δ*_*s*_ and *δ*_*w*_. Thus the parameter optimization strategy for CRBF is of great importance. The SVM parameters are traditionally determined by empirical means or via cross-validation. The empirical method is highly subjective and practically infeasible. The cross-validation approach is prone to getting stuck in a local minimum and it is hard to achieve the optimal approximation of a function because it requires the function to be continuous and derivable. In recent years, particle swarm optimization (PSO) algorithms and genetic algorithms (GA) have been used widely and effectively for SVM parameter selection and optimization.

**Figure 3:**
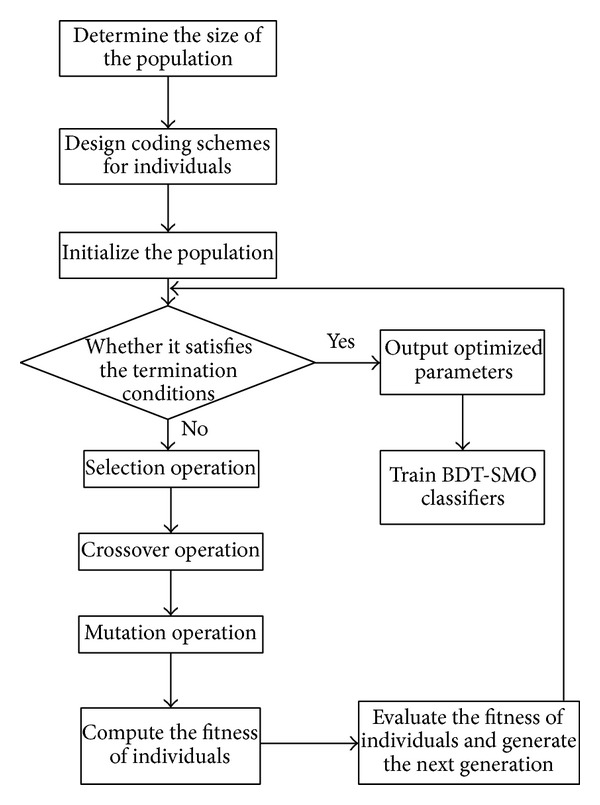
GA-based parameter optimization steps for BDT-SMO.

In this work, GA was first used to obtain the kernel parameters of SRBF (including parameters *C* and *δ*) and CRBF (including parameters *C*, *λ*, *δ*_*s*_, and *δ*_*w*_). Next, the trained BDT-SMO classifier is employed to classify the hyperspectral fused images of land usages. SRBF and CRBF parameters can be obtained via GA according to the following steps in Figure 3 [26]. CRBF has more kernel parameters than SRBF, and the parameters optimization processes of SRBF and CRBF are similar, so only the parameters' optimization process of CRBF based on GA is described below.

(1) The BDT-SMO training samples are taken as the original population, the population size is determined, binary encoding is performed on all parameters, and the original GA population is selected randomly.

**Figure 4:**
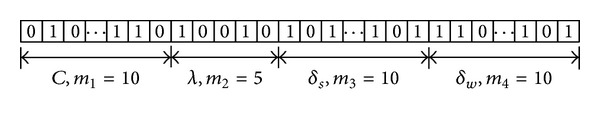
The structure of chromosomes.

In order to transfer the excellent gene segments to the next generation better, the corresponding chromosomes (a string composed of specific symbols according to a certain order) of the parameters (*C*, *λ*, *δ*_*s*_, and  *δ*_*w*_) should be designed by a coding mechanism before searching process of GA. In this work, the four parameters of CBRF are encoded with binary codes, and the order of parameters in chromosome is *C*, *λ*, *δ*_*s*_, and *δ*_*w*_. The lengths of the four parameters are represented with *m*_1_, *m*_2_, *m*_3_, and *m*_4_, respectively, and the length of the chromosome is *m*_1_ + *m*_2_ + *m*_3_ + *m*_4_, so the length of searching interval is 2^*m*_1_+*m*_2_+*m*_3_+*m*_4_^. In this work, the values of *m*_1_~*m*_4_ are set as follows: *m*_1_ = 10, *m*_2_ = 5, *m*_3_ = 10, and *m*_4_ = 10. The searching intervals of the parameters' optimization are set according to the possible value ranges of the parameters as follows: *C* ∈ [0,1000], *λ* ∈ [0.01,1], *δ*_*s*_ ∈ [0.01,10], and *δ*_*w*_ ∈ [0.01,10]. The structure of the chromosomes is shown in Figure 4.

(2) The fitness function is designed to measure how suitable the selected parameters are, and the fitness of each individual in the population is computed. In this work, the kernel parameters are optimized to improve the prediction accuracy of classification, that is, to make the actual values of the training samples approach the predicted values as closely as possible. Therefore, the total difference between the actual values and the predicted values is a proper measurement of the fitness. The fitness function used in this work is shown as follows [19]:

(15) $$\begin{array}{c} F\left(C,\lambda ,\delta_{S},\delta_{W}\right)=1-\sqrt{\frac{\sum_{i=1}^{n}{\left(y_{i}-y_{i}^{\prime }\right)}^{2}}{n}}. \end{array}$$

In (15), *n* represents the number of training samples and *y*_*i*_′ and *y*_*i*_ represent the prediction value and actual value of the training sample *i*. The bigger the *F* value is, the better the fitness of the corresponding parameters becomes. That is, the quality of the individuals will be better, and the possibility that better genes are inherited to the next generation will be greater.

(3) Independent genetic manipulation is performed on the population: the independent genetic manipulation in this paper includes selection, crossover, mutation, and fitness evaluation. Firstly, the fitness of all individuals is calculated and the roulette wheel selection strategy is adopted to select individuals from the current population for evolution into the next generation. Secondly, a crossover operation is performed using the one-point crossover algorithm and the crossover probability (*P*_*c*_) to obtain new individuals for the next generation [27]. Thirdly, a mutation operation is performed using the simple mutation algorithm and the mutation probability (*P*_*m*_); new individuals are generated by randomly changing some gene segments in the old individuals. Finally, in the fitness evaluation, the fitness function (15) is used to measure the merits and drawbacks of the individuals. In the genetic algorithm, designing the appropriate fitness function can improve the optimization efficiency. In this work, the population size of GA is 100 and the values of *P*_*c*_ and *P*_*m*_ are as follows: *P*_*c*_ = 0.8, *P*_*m*_ = 0.20.

(4) Check whether it satisfies the termination conditions. If it does, the GA is terminated and the optimal parameters are returned. Otherwise, the third step is repeated to generate new parameter values. When the times of iterations reach the specified number or the satisfied solutions have been obtained, the iterations will be terminated and the results will be outputted. In this work, in order to avoid too long search time, not more than 1000 times of iterations are taken to get the final optimized parameters unless the satisfied values of the four parameters have been obtained.

## 7. Experiments and Analysis

### 7.1. Experimental Zones and Samples Selection

#### 7.1.1. Experimental Zones and Related Images

To support the credibility of the experimental results, two zones are used in the experiment. The first zone is located in Xindian Village, Jinan District, part of Gulou District, Fuzhou, adjacent to Fuzhou National Forest Park. The covered area forms a rectangular shape, and the positions of the four apexes are (N26°10′24.87′′, E119°17′40.90′′), (N26°10′22.31′′, E119°20′19.64′′), (N26°6′39.45′′, E119°17′36.49′′), and (N26°6′36.90′′, E119°20′15.14′′). The zone has an elevation of 5~500 m, where the northern terrain is higher than the south, and the north is mostly mountainous with an elevation of 50~590 m, covered by laurel forest, mason pine, and tea garden, and interlaced with lanes, shrub-meadow, and bare land. The south part is flat as the core of the urban area of Fuzhou. The EO-1 Hyperion hyperspectral image and the EO-1 ALI panchromatic image for this zone are of the size 155 by 230 pixels and 465 by 690 pixels, respectively, and were captured on March 26th, 2003, covering an area of about 32.09 km^2^.

The second zone is located in Huangyan District, Taizhou, Zhejiang, and is part of the major urban area in Taizhou. The covered area takes a rectangular shape, and the positions of the four apexes are (N28°40′40.14′′, E121°14′55.85′′), (N28°40′42.20′′, E121°17′37.17′′), (N28°37′22.34′′, E121°14′59.14′′) and (N28°37′24.40′′, E121°17′40.37′′). The southeastern part of the zone is mostly mountain and forest land with an elevation of 50~500 m, covered by laurel forest, mason pine, and orchard, and interlaced with shrub-meadow, bare land, and lanes. As the heart of the urban area of Fuzhou, other parts of the zone are flat with an elevation of 5~50 m, rich in buildings, farmland, roads, and a small quantity of woodland and rivers. The Hyperion hyperspectral image and the ALI panchromatic image for this zone are 153 by 214 pixels and 459 by 642 pixels, respectively, and were captured on March 10th, 2003, covering an area of about 29.47 km^2^.

**Figure 5:**
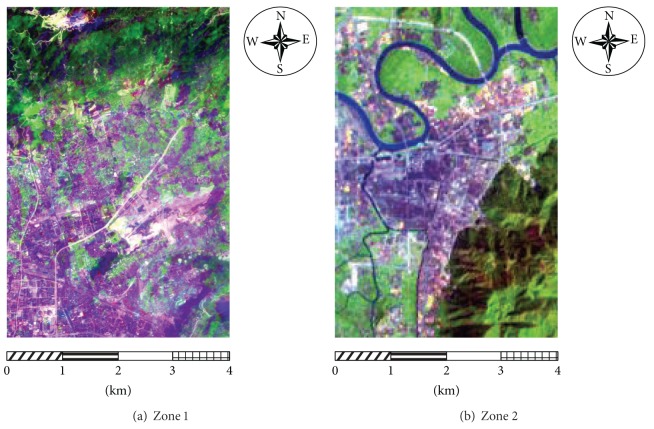
Results of fusing images in the two zones.

The obtained Hyperion hyperspectral images and the ALI panchromatic images needed preprocessing (e.g., remove undesired bands (the original images contain 241 bands), convert radiation values, remove bad lines, repair stripes, eliminate the Smile effect, perform atmospheric correction, geometric accuracy correction, etc.). The aim is to generate Hyperion images with 134 ground reflectance bands and ALI images with 1 panchromatic band. Next, the Gram-Schmidt (GS3) method is adopted to fuse the images above. The images are fused very effectively because the Hyperion and ALI images are both from the EO-1 sensors and have the same phase. In addition, existing research [28] has shown that, compared with other methods of hyperspectral images fusion, Gram-Schmidt (GS3) method can both maintain the spectral features and spatial texture features of hyperspectral images, and it is a relatively ideal method for the fusion of Hyperion hyperspectral images and ALI panchromatic images. The fused images from the two zones are shown in Figure 5.

The land in the two zones is divided into 12 classes based on its usage: farmland (C1), forest (C2), garden (C3), grassland (C4), bare land (C5), industrial and mining warehousing land (C6), residential land (C7), public administration/commerce and service/land concerning foreign affairs (C8), special land (C9), communications and transportation land (C10), water space (C11), and others (C12).

#### 7.1.2. Selection of Samples

Representative regions are selected through field work and comprehensive analysis of remote sensing images to collect ground-object samples. It is usually required that the training samples are no fewer than the number of test samples and the training samples for each type of ground object are no fewer than the number of classification features. With the assistance of the high resolution images of the zones and the 1 : 10000 land usage maps, all the representative samples are extracted for each class of land usage from the two experimental zones, as shown in Tables 1 and 2.

After the samples are extracted, it is required that the feature data of the training and test samples are normalized, in order to eliminate the difference of features in terms of dimension, order of magnitude, and data dispersion.

### 7.2. Extraction and Selection of Classification Features

Because some ground objects (e.g., roads, buildings, mountains, and rivers) in the experimental zones show highly obvious textural or terrain features, the classification features in this work include the spectral, textural, and terrain features. To improve classification accuracy, as many features as possible are encompassed in this work because the overfitting problem in SVM schemes is not serious when the number of the features is larger.

One hundred and eighty-one features are extracted from the images of the experimental zones, including 136 spectral features, 42 textural features, and 3 terrain features. The spectral features include 134 ground reflectance features, 1 NDVI feature (normalized vegetation index), and 1 NDBI feature (normalized construction index). The textural features involve 6 gray distribution features (second moment, contrast, correlation, variance, inverse gaps, and information entropy extracted through the use of the joint occurrence gray matrix method), 15 gray gradient features [29] (e.g., small gradient dominance, large gradient dominance, heterogeneity of gray distribution extracted via the gray-gradient joint occurrence matrix method proposed by Hong Jiguang in 1984), and 21 textural scale features (semivariance, energy, and mean extracted from the 7 high-frequency components after the second level wavelet decomposition). The terrain features include 3 features (elevation, slope, and aspect) extracted from the DEMs of the experimental zones.

All the features belonging to different types can be represented by different feature vectors as follows:

 spectrum: ST = [ST_1_, ST_2_,…, ST_134_, ST_NDVI_, ST_NDBI_];
 texture (gray-distribution): *F* = [*F*_1_, *F*_2_,…, *F*_6_];
 texture (gray-gradient): *T* = [*T*_1_, *T*_2_,…, *T*_15_];
 texture (gray-scale): *W* = [*W*_1_, *W*_2_,…, *W*_21_];
 terrain: *D* = [*D*_elev_, *D*_slp_, *D*_asp_].

The 5 feature vectors above can be integrated into 16 different combined feature vectors; the details of the different combined feature vectors are shown in Table 3.

In the classification experiment of remote sensing images, the test samples are used to verify the accuracy of the classification, and the classification results can be analyzed through the confusion matrixes. The evaluation indexes of classification results are commonly user accuracy (UA), producers' accuracy (PA), and overall accuracy (OAA) and Kappa coefficient. In addition, the speed of the classification can be measured by the prediction time (TS). In this work, in order to select the best classification method and feature vector, some cross-experiments are performed, respectively, with the 16 different feature vectors above and 5 different classification methods. The classification methods include the minimum distance classifier (MDC), maximum likelihood classifier (MLC), BP neural network (BPN), spectral angle mapping (SAM), and sequential minimal optimization (SMO).

**Figure 6:**
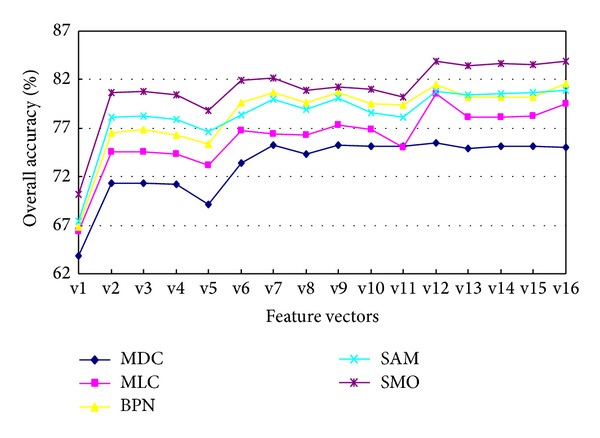
The effect on overall classification accuracy by different feature vectors and classificationmethods (experimental zone 1).

**Figure 7:**
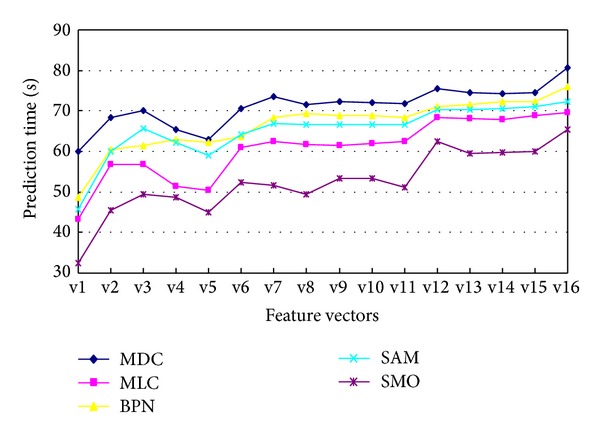
The effect on classification prediction time by different feature vectors and classification methods (experimental zone 1).

In order to analyze the influence of different numbers of training samples on the classification results, seven different numbers of sample subsets are selected randomly from the samples extracted from the experimental zones. The seven subsets relatively contain 5%, 10%, 20%, 40%, 60%, 80%, and 100% of the extracted samples and are applied to classification experiments using SVM and other methods. The stability of the classification results can be measured by the standard deviation. Obviously, each classification set has been repeated 7 times. The experiments are relatively repeated 7 times with different number of samples, and the classification results in Figures 6 and 7 are the stable values in the seven experiments. The experimental results are shown in Figures 6 and 7 (because the classification results of the two experimental zones are very similar, so only the result of experimental zone 1 is shown below).

Figures 6 and 7 show that the accuracy of the classification methods based on only spectral feature vector (v1) is lower than the other feature vectors, and the accuracy and speed of SMO method are higher than the other methods for the all feature vectors. In addition, the accuracy of SMO is the highest of all using the feature vector of v12 (i.e., ST + *F* + *T* + *W*).

In addition, the different combined feature vectors have the similar effects on the Kappa coefficient and the overall accuracy, so the effect on the latter will not be shown here.

Therefore, in the land use classification of hyperspectral fused images, the method of SMO based on the features (178 dimensions) combined of spectrum and textural features can achieve the best classification accuracy and speed.

### 7.3. SMO-Based Multiclass Classification of Hyperspectral Fused Images of Land Usage

It is first required that the optimal SMO-based multiclass classification approach is determined before classification using the combined kernel-based SMO method which is undertaken. The training samples of the two zones are learned using OAO-SMO, OAA-SMO, DAG-SMO, ECC-SMO, and BDT-SMO, respectively, based on the same kernel (SRBF). GA is used to optimize the kernel parameters *C* and *δ*. The experiments are relatively repeated 7 times with different number of samples like in Section 7.2. The seven subsets relatively contain 5%, 10%, 20%, 40%, 60%, 80%, and 100% of the extracted samples and are applied to classification experiments using different SMO multiclass classification approaches. The stability of the classification results can be measured by the standard deviation. The experimental results are provided in Tables 4 and 5, and the classification accuracies in tables are all the stable values in the seven experiments.

From Tables 4 and 5, the overall accuracy, average accuracy, Kappa coefficient values, and standard deviation of BDT-SMO approach are all higher than the other approaches. Obviously, BDT-SMO is the best multiclass classification approach for hyperspectral fused images.

### 7.4. CRBF-Based BDT-SMO Classification of Hyperspectral Fused Images of Land Usage

Land usages of the two zones are classified using linear, polynomial, sigmoid, SRBF, and CRBF-based BDT-SMO classifiers, respectively. The experiments are relatively repeated 7 times with different number of samples like in Section 7.3. The seven subsets relatively contain 5%, 10%, 20%, 40%, 60%, 80%, and 100% of the extracted samples and are applied to BDT-SMO classification experiments using different kernel functions. The stability of the classification results can be also measured by the standard deviation. The experimental results are provided in Tables 6 and 7, and the classification accuracies in tables are all the stable values in the seven experiments.

Tables 6 and 7 deliver the experimental results of the two zones, where *r* and *d* denote the offset coefficient and the number of polynomial orders, *v* and *w* denote the scale and attenuation parameters of the sigmoid kernel, and GA are employed to optimize the kernel parameters.

**Figure 8:**
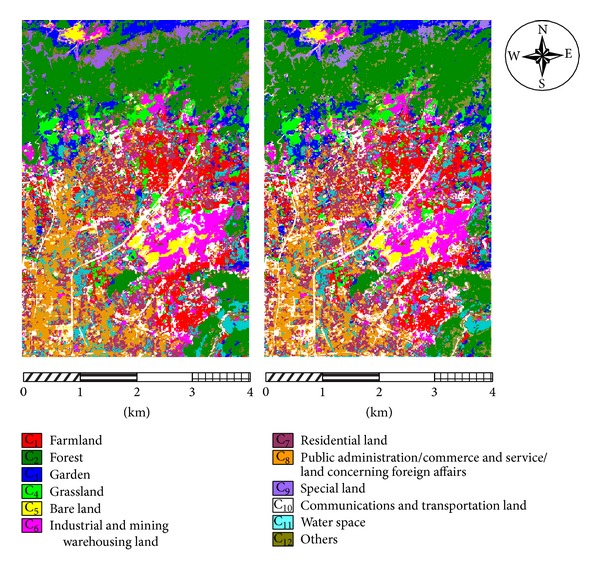
Comparison of BDT-SMO classification based on SRBF and CRBF (zone 1).

**Figure 9:**
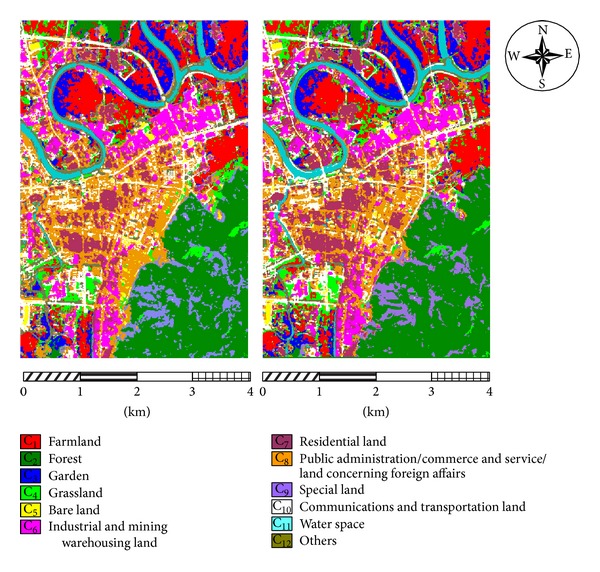
Comparison of BDT-SMO classification based on SRBF and CRBF (zone 2).

From Tables 6 and 7, it can be seen that the CRBF-based BDT-SMO classifiers have the highest overall accuracy (at 85.43% and 86.15%, 1.97% and 2.10% higher than the SRBF kernel), average accuracy (at 85.67% and 85.52%, 2.01% and 2.15% higher than the SRBF kernel), and Kappa coefficient values (0.8411 and 0.8483). The standard deviations of the classification accuracies of the CRBF-based BDT-SMO classifiers (0.63 and 0.62) are obviously lower than the others. In addition, CRBF has the fastest training speed second only to SRBF and the fastest test speed. The standard deviations of the CRBF-based and SRBF-based BDT-SMO classifiers are very similar and obviously lower than the others. Although, compared with SRBF, the training speed of the CRBF-based BDT-SMO classifiers is a little lower than SRBF-based, but the CRBF-based BDT-SMO classifiers have the better accuracy and test speed in classification and have almost the same stability with the SRBF-based BDT-SMO classifiers. Apparently, CRBF-based BDT-SMO classifiers have the better performance. The results of the CRBF-based and SRBF-based BDT-SMO classification of hyperspectral sensing images of land usages are provided in Figures 8 and 9. In addition to the overall classification results, the accuracy of each individual class of land usages is also improved, as shown in Tables 8 and 9. From the tables above, it can be seen that CRBF can more accurately classify each class of land usage. Especially for the four difficult classes—C_3_ (garden), C_5_ (bare land), C_8_ (public administration/commerce and service/land concerning foreign affairs), and C_10_ (communications and transportation land), the classification accuracy is improved enormously, and the producers' accuracy (PA) is up to 80.04%–80.51%, 66.17%–67.84%, 83.13%–84.76%, and 82.54%–84.07%, which is 3.99%–5.93%, 2.26%–2.78%, 3.37%–5.21%, and 3.13%–4.44% higher than the single RBF kernel. Therefore, the CRBF-based BDT-SMO classifiers can classify the land usages using hyperspectral fused images in experimental zones more effectively.

To more intuitively show the benefit of CRBF for classification of C_3_, C_5_, C_8_, and C_10_, 4 instances are selected, respectively, from the two zones for verification. BDT-SMO classification results of these instances under the single RBF and CRBF are provided in Table 10.

From Table 10, it can be seen that the 8 positions are all misclassified when the SRBF-based BDT-SMO classifier is used and are all classified correctly when the CRBF-based BDT-SMO classifier is used. Therefore, the CRBF-based BDT-SMO classifier is excellent enough to classify land usage using hyperspectral fused images effectively and efficiently.

## 8. Conclusions

In order to improve the accuracy and efficiency of the classification of hyperspectral fused images, a new combined radial basis kernel function (CRBF) is proposed by combining spectral and textural features in a weighted manner. The binary decision tree-based multiclass SMO (BDT-SMO) based on the kernel function of CRBF is used in the classification of hyperspectral fused images, and the parameters of CRBF-based BDT-SMO classifiers are optimized by genetic algorithms (GA). Experimental results show that, compared with SRBF, CRBF-based BDT-SMO classifiers have better accuracy and efficiency in the classification of hyperspectral fused images.

## Figures and Tables

**Table 1:** All the samples extracted from the first zone.

Type of land usage	C_1_	C_2_	C_3_	C_4_	C_5_	C_6_	C_7_	C_8_	C_9_	C_10_	C_11_	C_12_
Number of training samples	608	528	838	538	706	616	648	630	604	1294	772	612
Number of test samples	1830	1558	2523	2621	2123	1853	1949	1895	1818	3887	2325	1841

**Table 2:** All the samples extracted from the second zone.

Type of land usage	C_1_	C_2_	C_3_	C_4_	C_5_	C_6_	C_7_	C_8_	C_9_	C_10_	C_11_	C_12_
Number of training samples	810	732	846	720	700	708	848	690	804	1220	806	688
Number of test samples	2435	2203	2540	2163	2105	2127	2545	2073	2415	3664	2423	2071

**Table 3:** The details of the different combined feature vectors.

Number	Combined feature vectors
v1	ST
v2	ST + *F*
v3	ST + *T*
v4	ST + *W*
v5	ST + *D*
v6	ST + *F* + *T*
v7	ST + *F* + *W*
v8	ST + *F* + *D*
v9	ST + *T* + *W*
v10	ST + *T* + *D*
v11	ST + *W* + *D*
v12	ST + *F* + *T* + *W*
v13	ST + *F* + *T* + *D*
v14	ST + *F* + *W* + *D*
v15	ST + *T* + *W* + *D*
v16	ST + *F* + *T* + *W* + *D*

**Table 4:** Comparison of classification results under different SMO multiclass classification approaches (zone 1).

Classification approaches	Overall accuracy (%)	Average accuracy (%)	Kappa coefficient	Training time (s)	Test time (s)	Standard deviation	Optimized parameters
OAO-SMO	82.43	80.06	0.7998	24.36	21.35	0.95	*C* = 10, *δ* = 0.075
OAA-SMO	81.09	79.89	0.7875	25.37	24.10	1.85	*C* = 10, *δ* = 0.070
DAG-SMO	81.25	81.29	0.7904	30.42	15.70	1.36	*C* = 15, *δ* = 0.050
ECC-SMO	82.05	82.34	0.7954	31.71	15.05	1.65	*C* = 10, *δ* = 0.550
BDT-SMO	83.46	83.66	0.8195	30.57	13.67	0.65	*C* = 15, *δ* = 0.055

**Table 5:** Comparison of classification results under different SMO multiclass classification approaches (zone 2).

Classification approaches	Overall accuracy (%)	Average accuracy (%)	Kappa coefficient	Training time (s)	Test time (s)	Standard deviation	Optimized parameters
OAO-SMO	81.57	81.79	0.7994	32.33	24.74	1.11	*C* = 10, *δ* = 0.080
OAA-SMO	81.21	80.75	0.7837	31.64	26.75	1.21	*C* = 10, *δ* = 0.075
DAG-SMO	82.31	80.79	0.8106	40.12	16.84	1.32	*C* = 15, *δ* = 0.055
ECC-SMO	82.52	81.32	0.8153	43.35	16.01	1.33	*C* = 10, *δ* = 0.550
BDT-SMO	84.05	83.37	0.8254	42.11	14.59	0.59	*C* = 15, *δ* = 0.065

**Table 6:** Comparison of BDT-SMO classification approaches under different kernels (zone 1).

Kernels	Overall accuracy (%)	Average accuracy (%)	Kappa coefficient	Training time (s)	Test time (s)	Standard deviation	Optimized parameters
Linear	79.34	79.39	0.7752	35.24	18.49	1.88	
Polynomial	80.05	80.41	0.7841	39.79	20.63	1.79	*r* = 1, *d* = 3
Sigmoid	82.12	82.55	0.8024	38.15	22.94	1.52	*v* = 20, *w* = 0.50
SRBF	83.46	83.66	0.8195	30.57	13.67	0.65	*C* = 15, *δ* = 0.055
CRBF	85.43	85.67	0.8411	33.27	12.44	0.63	*C* = 15, *λ* = 0.255 *δ*_*s*_ = 0.035, *δ*_*w*_ = 0.055

**Table 7:** Comparison of BDT-SMO classification approaches under different kernels (zone 2).

Kernels	Overall accuracy (%)	Average accuracy (%)	Kappa coefficient	Training time (s)	Test time (s)	Standard deviation	Optimized parameters
Linear	80.11	79.33	0.7841	43.15	16.41	1.85	
Polynomial	81.19	80.06	0.7859	43.59	22.79	1.75	*r* = 1, *d* = 3
Sigmoid	82.49	81.39	0.8009	44.95	15.29	1.61	*v* = 20, *w* = 0.55
SRBF	84.05	83.37	0.8254	42.11	14.59	0.59	*C* = 15, *δ* = 0.065
CRBF	86.15	85.52	0.8483	43.14	13.95	0.62	*C* = 15, *λ* = 0.255 *δ*_*s*_ = 0.045, *δ*_*w*_ = 0.055

**Table 8:** Land usage classification accuracy (zone 1).

Type of land usage	C_1_	C_2_	C_3_	C_4_	C_5_	C_6_	C_7_	C_8_	C_9_	C_10_	C_11_	C_12_
Zone 1 (%)	92.13	95.67	76.05	88.45	63.91	82.86	84.89	79.76	88.51	79.41	91.90	80.36
Zone 2 (%)	90.91	97.58	74.58	84.77	65.06	83.40	84.18	79.55	88.28	79.63	91.03	81.46

**Table 9:** Land usage classification accuracy (zone 2).

Type of land usage	C_1_	C_2_	C_3_	C_4_	C_5_	C_6_	C_7_	C_8_	C_9_	C_10_	C_11_	C_12_
Zone 1 (%)	93.70	99.78	80.04	89.44	66.17	82.16	86.15	83.13	89.08	82.54	93.48	82.34
Zone 2 (%)	92.38	99.57	80.51	85.55	67.84	82.21	84.32	84.76	89.24	84.07	92.03	83.71

**Table 10:** CRBF-based BDT-SMO classification examples.

Verification instance	Zone	Specific position	Right class	Classification results (SRBF)	Classification results (CRBF)
Instance 1	Zone 1	Near Jiao Mountain in the north of Jin'an District	C_3_	C_2_	C_3_

Instance 2	Zone 2	Between MaAnShan Village and WangLinYang Village and nearby	C_3_	C_1_	C_3_

Instance 3	Zone 1	Near Fontainebleau Community and the Residence Theme Park	C_5_	C_10_	C_5_

Instance 4	Zone 2	Near YaLin Village in the south Second Ring Road	C_5_	C_10_	C_5_

Instance 5	Zone 1	Near JianTianJiuDong Innovation Garden in the north of XiFeng Road	C_8_	C_10_	C_8_

Instance 6	Zone 2	Near the First Vocational and Technical College in HuangYan	C_8_	C_10_	C_8_

Instance 7	Zone 1	Section of the South FuFei Road between the Third Ring freeway and the North Ring Road, and the middle section of the North Ring Road	C_10_	C_8_/C_5_	C_10_

Instance 8	Zone 2	YongNingJiang Bridge (bridge floors of the East Second Ring Road, DaQiao Road and LuTing Road)	C_10_	C_12_	C_10_

## References

[1] Vapnik VN (1995). *The Nature of Statistical Learning Theory*.

[2] Vapnik VN (1998). *Statistical Learning Theory*.

[3] Burges CJC (1998). A tutorial on support vector machines for pattern recognition. *Data Mining and Knowledge Discovery*.

[4] Cortes C, Vapnik V (1995). Support-vector networks. *Machine Learning*.

[5] Foody GM, Mathur A (2004). Toward intelligent training of supervised image classifications: directing training data acquisition for SVM classification. *Remote Sensing of Environment*.

[6] Foody GM, Mathur A (2006). The use of small training sets containing mixed pixels for accurate hard image classification: training on mixed spectral responses for classification by a SVM. *Remote Sensing of Environment*.

[7] Xue N (2011). Comparison of multi-class support vector machines. *Computer Engineering and Design*.

[8] Do TN, Nguyen V-H, Poulet F (2009). GPU-based parallel SVM algorithm. *Journal of Frontiers of Computer Science and Technology*.

[9] Platt JC (1999). *Fast Training of support Vector Machines Using Sequential Minimal Optimization*.

[10] Keerthi SS, Shevade SK, Bhattacharyya C, Murthy KRK (2001). Improvements to Platt's SMO algorithm for SVM classifier design. *Neural Computation*.

[11] Dietterich TG, Bakiri G (1995). Solving multiclass learning problems via error-correcting output codes. *Journal of Artificial Intelligence Research*.

[12] Liu Y Using SVM and error-correcting codes for multiclass dialog act classification in meeting corpus.

[13] Ai Q, Qin Y, Zhao J (2011). An improved directed acyclic graphs support vector machine. *Computer Engineering and Science*.

[14] Platt JC, Cristianini N, Shawe-Taylor J (2000). Large margin DAGs for multiclass classification. *Proceedings of Advances in Neural Information Processing Systems*.

[15] Li K, Ren Q, Wen B (2010). Application of separation degree and binary decision tree-based multi-class SVM in classification and recognition of weld defects. *Journal of Sichuan University (Natural Science)*.

[16] Wang X, Qin Y (2008). Research on SVM multi-class classification based on binary tree. *Journal of Hunan Institute of Engineering*.

[17] Madzarov G, Gjorgjevik D Multi-class classification using support vector machines in decision tree architecture.

[18] Huang F (2014). Research on classification of hyperspectral remote sensing imagery based on BDT-SMO and combined features. *Journal of Multimedia*.

[19] Tooke TR, Coops NC, Goodwin NR, Voogt JA (2009). Extracting urban vegetation characteristics using spectral mixture analysis and decision tree classifications. *Remote Sensing of Environment*.

[20] Wang G (2006). Properties and construction methods of kernel in support vector machine. *Computer Science*.

[21] Camps-Valls G, Gomez-Chova L, Muñoz-Marí J, Vila-Francés J, Calpe-Maravilla J (2006). Composite kernels for hyperspectral image classification. *IEEE Geoscience and Remote Sensing Letters*.

[22] Camps-Valls G, Bruzzone L (2005). Kernel-based methods for hyperspectral image classification. *IEEE Transactions on Geoscience and Remote Sensing*.

[23] Benoudjit N, Verleysen M (2003). On the kernel widths in radial-basis function networks. *Neural Processing Letters*.

[24] Chapelle O, Vapnik V, Bousquet O, Mukherjee S (2002). Choosing multiple parameters for support vector machines. *Machine Learning*.

[25] Feng G (2011). Parameter optimizing for Support Vector Machines classification. *Computer Engineering and Applications*.

[26] Huang C, Wang C (2006). A GA-based feature selection and parameters optimizationfor support vector machines. *Expert Systems with Applications*.

[27] Zhong M (2011). Research on intelligent schedule of public traffic vehicles based on parallel genetic algorithm. *Computer Era*.

[28] Huang F, Yan L (2013). Study on the hyperspectral image fusion based on the gram_schmidt improved algorithm. *Information Technology Journal*.

[29] Hong J (1984). The texture analysis methods of gray gradient co-occurrence matrix. *Acta Automatica Sinica*.

